# Laryngeal Schwannoma: Case Report and Literature Review

**DOI:** 10.5402/2011/540643

**Published:** 2011-05-31

**Authors:** Y. Ramakrishnan, W. J. Issing

**Affiliations:** ENT Department, Freeman Hospital, Freeman Road, High Heaton, Newcastle upon Tyne NE7 7DN, UK

## Abstract

Neurogenic tumours of the larynx, particularly schwannomas are rare. We report a case report of a schwannoma in a 30-year-old woman which was excised endoscopically. The aim of this paper is to highlight this rare condition and management options to the otolaryngological community.

## 1. Case Report

A 30-year-old lady presented to the outpatient clinic with progressive dysphonia and exertional dyspnea over a 2-year period. There were no associated systemic symptoms. She was a nonsmoker and consumed alcohol occasionally. On examination, she was dysphonic but not stridulous or dyspneic. Flexible nasoendoscopy revealed a soft tissue swelling in the supraglottic region starting from the posterior commissure blocking the view of the vocal cords. Computer tomography (CT) showed a well circumscribed 1.5 × 1.4 cm low attenuation midline supraglottic mass, commencing just below the aryepiglottic folds and extending onto sclerotic arytenoids (Figures 3 and 4). The attenuation of this mass was slightly greater than that of fluid, suggesting this could represent a retention cyst containing proteinaceous secretions. 

**Figure 1:**
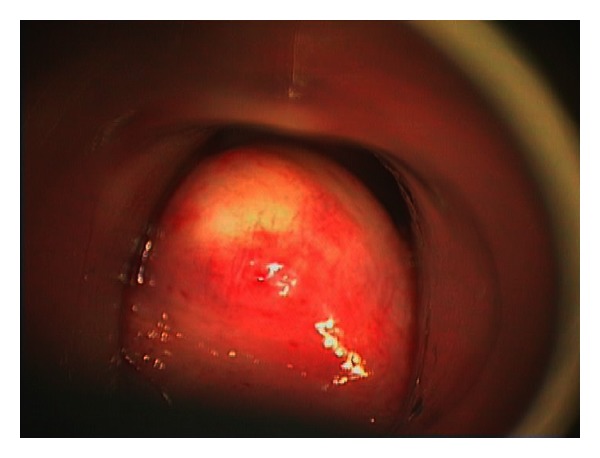
Mass presenting as a submucosal bulge obstructing view of vocal cords.

**Figure 2:**
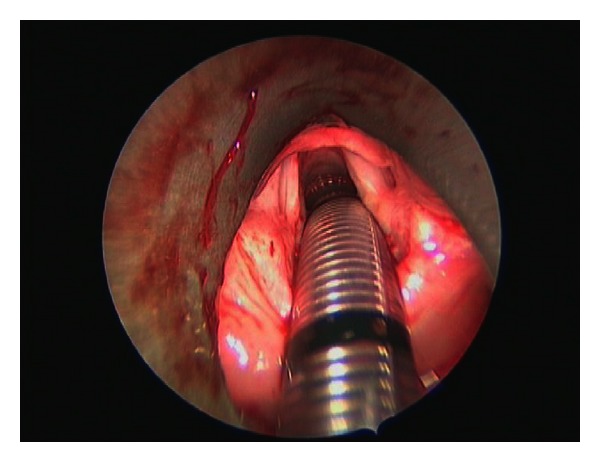
Postoperative view following complete excision of the schwannoma.

**Figure 3:**
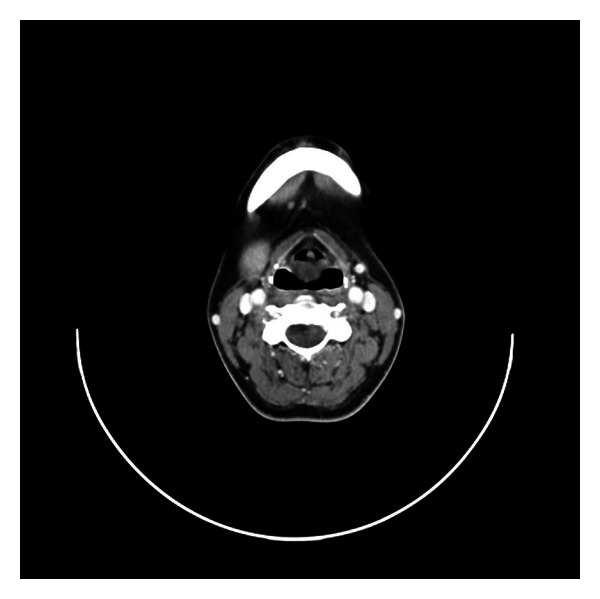
Serial CT axial images of the midline supraglottic mass commencing just below the aryepiglottic folds and extending onto arytenoids.

**Figure 4:**
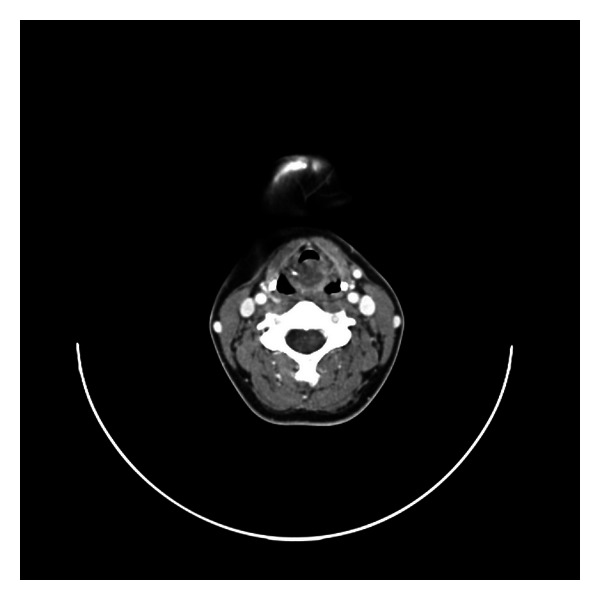
Serial CT axial images of the midline supraglottic mass commencing just below the aryepiglottic folds and extending onto arytenoids.

Suspension microlaryngoscopy and biopsy was performed. Intraoperatively, a solid, well-encapsulated lesion was shelled out using cold steel instruments (Figures 1 and 2). The patient was extubated postoperatively and did not require a tracheostomy. Histopathological examination confirmed the presence of an arytenoid schwannoma. 3 months following surgery, no recurrence was evident.

## 2. Introduction

Schwannomas are benign slow-growing tumours that arise from the Schwann cells of any nerve (peripheral, cranial, or autonomic). Those affecting the head and neck are commonly intracranial (e.g., vestibular nerve) or parapharyngeal (arising from glossopharyngeal, vagal, accessory, hypoglossal nerve, and sympathetic chains). Neurogenic tumors of the larynx (e.g., schwannoma or neurofibroma) therefore remain extremely rare. Indeed, it represents less than 1.5% of all benign tumours (the majority being papillomas and chondromas) [1]. It is a slow, growing encapsulated tumour commonly presenting in the 4th and 5th decade of life, especially women. However, it can present at any age. Over 130 cases have been reported in the worldwide literature since it was first described in 1925 [2].

The origin of the schwannoma is presumed to be in the internal branch of the superior laryngeal nerve [3]. Often however, this is not discernible intraoperatively and likely to originate from the smaller distal nerve fibres in the laryngeal submucosa. The most common anatomical site is the aryepiglottic fold, followed by the arytenoids, ventricular folds, and vocal cord [4, 5]. The risk of malignant transformation of laryngeal schwannoma is very rare [6].

From a clinical perspective, it is vital to differentiate a schwannoma from a neurofibroma for 2 reasons. Firstly, neurofibromas have a higher potential of recurrence and malignancy (approximately 10%). Secondly, the presence of neurofibromas should also alert the physician to the exclusion of neurofibromatosis. The definitive diagnosis is established histologically.

Patients typically present with dysphonia, stridor, dyspnea, dysphagia, globus sensation, or lateral neck lump. On laryngoscopy, most lesions appear as a smooth submucosal lesion, usually confined to the aryepiglottic fold or false vocal cord. It may obstruct the view of the laryngeal inlet and result in reduced mobility of the vocal cord. The latter may be due to “pseudofixation” of the cricoarytenoid joint as a result of mass effect of the lesion [5]. Almost all neurogenic tumors of the larynx arise from the supraglottis, the true vocal cord representing a rare origin site [7]. 

CT and MRI are valuable in defining the nature and extent of the lesion, with MRI offering superior soft tissue delineation. Typically, the lesion is sharply demarcated, round or oval, isoattenuated with muscle, and often heterogeneously enhanced [8]. Calcification which reflects degenerative change, though rare, has been reported in ancient schwannomas. Schwannoma and neurofibromas cannot be distinguished radiologically due to similar findings. The differential diagnosis includes laryngeal cyst and internal laryngocele.

The definitive diagnosis is performed histologically. Enger and Weiss established three histological criteria for the diagnosis of schwannoma: encapsulation, presence of Antoni A and/or Antoni B stroma, and S-100 protein positivity [9]. In Antoni A (cellular region), the spindle-shaped Schwann cells are compactly arranged with nuclei occasionally lining up in palisades to form Verocay bodies. Antoni B (less cellular) describe loosely arranged spindle Schwann cells within a myxoid matrix. In contrast, neurofibromas are unencapsulated and comprise a variety of cell types: elongated spindle Schwann cells interwoven with axons and collagen fibres. An important feature is that schwannoma grows extrinsic to the nerve fibre whilst in neurofibroma, the tumour is entwined with the parental nerve fascicles [5]. 

Surgery forms the mainstay of treatment of laryngeal schwannoma. A tracheostomy may be required to secure the airway. The surgical approach depends on the size and location of the lesion. Smaller lesions can be approached endoscopically with or without a laser. Larger tumours may require an external approach, for example, lateral thyrotomy, lateral pharyngotomy, or laryngofissure technique [10]. Wide excision is necessary to prevent recurrence. Rapid regrowth can occur within months following incomplete resection of the schwannoma [5]. Following surgery, restoration of vocal cord mobility has been reported independent of the approach [11, 12].

## 3. Conclusion

Despite its rarity, neurogenic tumors of the larynx need to be recognised. The primary management involves securing the airway. Despite various imaging modalities, the distinction between Schwannoma and neurofibromas can only be made histologically. 

Complete resection of the lesion is necessary to prevent recurrence.
